# Observations on the Prevention and Cure of Hydrophobia

**Published:** 1820-03

**Authors:** C. W. Smerdon

**Affiliations:** Member of the Royal College of Surgeons.


FOR THE LONDON MEDICAL AND PHYSICAL JOURNAL.

### *Observations on the Prevention and Cure of Hydrophobia.* By C. W. Smerdon, Member of the Royal College of Surgeons.

IF the old adage, "prevention is better than cure," be applicable to diseases in general, how much more so must it be to those which are either in their nature absolutely incurable, or are relatively so with regard to our imperfect knowledge of the healing art. The annals of medicine indeed furnish us with examples of recovery from every disease to which human nature is liable; but experience unfortunately teaches us, that extraordinary instances of cure should rather be ascribed to extraordinary efforts of nature in each individual case, than to the remedies, however judiciously employed. These solitary examples, therefore, do not lessen the value of preventive remedies, but on the contrary stimulate us to adopt them whenever they are rational and proper.

These introductory remarks are applicable to a disease, which, in the opinion of many, has uniformly proved fatal,—I mean hydrophobia. Nor is this certain termination the worst part of it: the dreadful sufferings of the unfortunate patient would excite pity even in the breast of the most hardened savage; nor would it be contrary to the dictates of humanity, were we to shorten the duration of his sufferings by means which, if they did not cure the disease, would promptly lead to the destruction of life. Happily for mankind, this disease occurs but seldom; and we have a preventive for it, which, when timely had recourse to, has never been known to fail. At first sight, the knife appears a dreadful alternative; and it is as much owing perhaps to the vacillating timidity of the surgeon, as to the well-grounded fears of the patient, that cauteries have been substituted for it; which, in some instances at least, have failed, and the lives of human beings consequently sacrificed. Such cases of failure, in which the consequences are certain death, are deserving of our most serious consideration. The surgeon who acts on his own responsibility ought to reflect that, on what he is about to do depends the certain recovery, or certain destruction, of his patient; and in no instance should he substitute an uncertain remedy, where the knife can with safety be had recourse to. I

do not mean to contend that there are no well-attested cases on record, of prevention after the use of cauteries only; but we have undoubted evidence of their failure in several cases* that have been recently published, notwithstanding, in two of them, they were perseveringly and largely applied: whereas, had the bitten part been excised, no doubt I think can be entertained, but that the lives of those patients would have been preserved. This inference is unpleasant; but it naturally grows out of such cases where the failure of a supposed antidote inevitably leads to the destruction of life.

But the bite of a rabid animal may be inflicted on a part which, from its situation, cannot be excised; and it now would become of considerable importance to decide, whether the caustic should be resorted to on the good old-woman principle,—that, if it be of no service, it cannot injure; or avoided, as being more likely to induce the disease than to prevent it, by exciting an increased action in the absorbents of the bitten part. Analogy strongly favours the latter proposition; and, if only a well-grounded doubt on that principle can be brought forward against the practice, it ought never to be had recourse to.

There are three ways by which poisons which are applied to the surface, are supposed to enter the system: 1st, by the absorbents; 2dly, by the nerves; and 3dly, by the veins. We have undoubted proof that the syphilitic poison is carried into the constitution by the first of these. The appearance of a chancre is peculiar to itself, and consequently must be produced by a peculiar irritant. A venereal bubo also possesses specific properties, which marks its nature to be different from glandular inflammation arising from common irritation: if no mercury be given, the gland bursts, and, having discharged its contents, takes on the characteristic properties of the primary sore; hence we learn, that the venereal poison is carried into the constitution through the medium of the absorbents.

It is more than probable, that all the classes of poisons which are applied to the cutis pass into the system by different routes; that the morbid poisons, all of which require to remain dormant a certain time before their effects are manifested, pass through the medium of the absorbents. The vegetable poisons, the effects of which are immediate, exert their influence on the nervous system and brain; and thus, when sufficiently concentrated, as for instance the woorara and a few others, destroy life very quickly, and with very little pain, by acting upon the sensorium. The animal poisons first induce a violent and destructive inflammation on the part to which they are applied,

* See Mr. Bellingham's case, published in this Journal; the case of a boy, that occurred some years ago at Lewes; also the last case lately published by Dr. Pinckard; and one in the November Number of the Repository.

and spreading rapidly along the cellular tissue, pass to the vital parts by direct communication.

We have numerous examples to prove that pain, the effect of irritation, always excites the actions of the absorbents: hence we apply stimulants to the eye, for the removal of one sort of opacity of the cornea. The disease of the bursæ called a ganglion, is best cured by whatsoever causes an inflammatory action within the sac. And we know that the matter contained in encysted tumors, or in indolent abscesses, becomes frequently absorbed, if by any means inflammation can be excited within them; in short, it may be assumed as a general, if not an universal, law of the animal economy, that the irritation of nerves, as evinced by pain, increases the action of the absorbent vessels.

Benjamin Bell, considering that chancre in the first instance is a local affection, thought that he could stop the further progress of the disease by destroying the surface of the primary sore with caustic; but, in almost all the cases which he thus treated, venereal buboes were the result, which ran rapidly into a state of suppuration: whereas, in most of those cases in which the system was immediately saturated with mercury without any local application, the venereal virus was subdued without any further extension of it. Thus he was satisfied, that the application of caustic to a venereal chancre, instead of destroying the disease, caused its rapid extension, by stimulating the action of the absorbent principles. Now, if these deductions be true with respect to one morbid poison, can it be philosophical to suppose that it is not so with respect to another? or, if the virus on a small venereal sore could not be destroyed by caustic, but on the contrary appeared to be rendered more active by it, is it not highly probable that the same law holds good with respect to the poison of rabies? I am convinced that no well-grounded argument can be adduced in favour of a contrary opinion. To cauterise therefore a bitten part which can be excised, would be deserving of the severest reprehension; and even the propriety of adopting this mode of treatment at all, in cases where the knife cannot be used, is, to say the least of it, extremely doubtful, and therefore is a question well deserving the attention of the faculty.

It is impossible to read the cases of hydrophobia which have been recently published by Dr. Pinckard, without feeling the deepest melancholy. Drawn up as they are in a manner that reflects the highest honour both to the head and to the heart of the writer, at the same time that they confirm him to be a man of considerable feeling and reflection, it is painful to perceive, that not the least progress was made either towards a mode of cure, or towards elucidating the true seat of the disease. If, however, we cannot tell what hydrophobia is, enough has been written to tell us what it is not. The dissections and treatment of these cases, as well as of others which have been published at different times, must convince every impartial reader, that visceral inflammation does not form the basis of that dreadful malady. The first symptom of hydrophobia has nothing to characterise it from the slight febricula which always accompanies a common cold. Sometimes indeed there is pain and redness in the bitten part, extending upwards in the course of the nerves or absorbents; but this, I believe, is not a uniform symptom. The attention of the patient is first awakened by an unaccountable sensation on the sight of a fluid: this, in the first instance, is very slight, and goes off entirely when the offending cause is removed. Now it is well worthy of remark, that it is either the *sight* of the fluid, or the mere desire for drink, *not the act of drinking*, which occasions the spasmodic rigor; hence then it is clear, that the impression is first made on the mind, and from thence, through the medium of the nerves, to the supposed seat of the disease. This incontrovertible fact is important, because it points out a clear distinction between hydrophobia and inflammatory diseases affecting the same parts. In cynanche laryngea, the patient is in danger of suffocation whenever deglutition is attempted, and he dreads the act of swallowing just as much as a person in hydrophobia. But, mark the distinction: in the one it is the *act* which produces the constriction, whilst in the other it is the *mind*, through the medium of the nerves. Hence I think it is evident, even to demonstration, that hydrophobia is an affection of nerves, principally, if not wholly, of the eighth pair and the grand sympathetic,* which (a certain impression being previously made on the mind) occasion in the first instance a spasmodic constriction only of those parts which they supply on their first exit from the skull. It would far exceed the necessary limits of this paper, were I to endeavour to prove that the extreme branches of nerves may be in a state of diseased action independent of the brain, as well as those of arteries independent of the heart. That the ultimate branches of arteries are disordered in their action, whenever the secretion which they produce is unhealthy, cannot, I think, be doubted. Why then is it irrational to suppose that the branches

* It would seem, from the situation of these two remarkable nerves, that they are intimately connected both in their structure and use. In man, and in the superior animals which are immediately in the chain beneath him, the par vagum and sympathetic unite on their exit from the skull, and again in the abdomen, to form the visceral nerve, and frequently in the last cervical ganglion. In the inferior animals which are warm-blooded the sympathetic is lost, and the par vagum supplies its place by traversing the body and uniting with the other nerves. Hence I conceive, that the functions of the one cannot be diseased without the other participating in it.

of nerves, which are governed by similar laws, are liable to similar diseases. With respect to the subsequent symptoms of hydrophobia, they are unquestionably the effect of a morbid sensibility of nerves, extended over the body through the medium of the sympathetic.

In all the experiments of Dr. Wilson Philip, and others, laborious respiration, almost amounting to suffocation, was the effect of a division of the eighth pair of nerves. "In all the instances," says Dr. Philip, "in which these nerves were divided, great dyspnœa soon came on, and the air-cells and tubes were found clogged with frothy mucus." Are not these affections strikingly similar to those which affect the unfortunate sufferer in hydrophobia? Does not a collection of thick viscid mucus about the pharynx, &c. form one of the most distressing of his symptoms? If this analogy be allowed, the same remedy which Dr. Philip founded on the above phenomena for asthma, might be applied to hydrophobia,—I mean galvanism. "From the fact," says Dr. Philip, "that oppressed breathing and the collection of phlegm occasioned by a division of the eighth pair of nerves were prevented, by sending a stream of galvanic gas through the lungs, I was induced to try its effects in habitual asthma, and obtained uniform relief. Two thin plates of metal about two or three inches in diameter, dipped in water, were applied, one to the nape of the neck, and the other to the pit of the stomach or rather lower. The wires from the different ends of the trough were brought into contact with these plates, and as great a galvanic power maintained as the patient could bear without complaint. In this way the galvanic influence was sent through the lungs as much as possible, in the direction of their nerves."*

This remedy may be as futile as every other which has been tried hitherto for hydrophobia. Notwithstanding, it is well worthy of a fair trial: 1st, because it is novel; 2dly, because its use does not exclude the administration of other means; and lastly, besides being a remedy for asthma, it has lately been found of singular efficacy in some of the most unpromising cases of epilepsy.

*Clifton, Bristol; December,* 1819.

---

* Philip on the Laws of the Vital Functions, &c.

